# Visual recursion without recursive language? a case study of a minimally verbal autistic child

**DOI:** 10.3389/fpsyt.2025.1540985

**Published:** 2025-06-23

**Authors:** Joana Rosselló, Alexandre Celma-Miralles, Mauricio Dias Martins

**Affiliations:** ^1^ Department of Catalan Philology and General Linguistics, University of Barcelona, Barcelona, Spain; ^2^ Center for Music in the Brain, Aarhus University & Royal Academy of Music Aarhus/Aalborg, Aarhus, Denmark; ^3^ Center for Brain and Cognition, Pompeu Fabra University, Barcelona, Spain; ^4^ SCAN-Unit, Department of Cognition, Emotion, and Methods in Psychology, Faculty of Psychology, University of Vienna, Vienna, Austria

**Keywords:** recursion, iteration, minimally verbal autism, hyperlexia, visual patterns

## Abstract

The human faculty to generate an infinite set of structured expressions in language, present in most cultures and normal ontogeny, is the most substantial evidence of the human capacity for recursion. In contrast, strong evidence of this capacity in other domains has been sparse, inviting the speculation that recursion is primarily linguistic and co-opted into other domains. Here, we present a case report of a minimally verbal 11-y.o. autistic child with poor language comprehension whose speech rarely exceeds two-word commands despite remarkable hyperlexia (i.e., mechanical reading in Spanish, Catalan, and English) and a visually-based, mainly nominal lexicon acquired through reading. Importantly, medium-range scores in visual tasks and hyperlexia suggest that he can detect complex visual patterns despite low fluid intelligence. Against this background, we tested whether this child could represent recursive hierarchical embedding in vision, despite no evidence of it in language. We found that 1) his accuracy was above chance and 2) it was not significantly different from that of typically developing children. Accordingly, we suggest that a core capacity of recursion, interfacing with a sensory modality and a visuospatial conceptual system, is sufficient to process recursive patterns in vision. In contrast, linguistic recursion may require more complex sensorimotor and conceptual-intentional machinery.

## Introduction

The human Faculty of Language allows the generation of an infinite set of structured expressions out of a finite set of words and rules. This property is assumed to result from a recursive process that embeds expressions within other expressions (1), creating complex hierarchical objects. The property of representing recursive hierarchical embedding (henceforth recursion) has been claimed in various domains (1–7). A crucial question remains whether recursion is domain-general, multiply-domain-specific (one recursive capacity per domain), or primarily linguistic and then used in other domains through linguistic computations (8–10).

In typical development cases and most populations, recursion emerges in syntax (1, 11–13) and at the level of linguistic discourse (14–16). This widespread and effortless use has led to the hypothesis that recursion is primarily linguistic, with its use in other domains dependent on the human Faculty of Language (17). Recursion has also been proposed in the domains of action planning (18–21), visuospatial processing (22), Theory of Mind (ToM) (23, 24), and music (25, 26). However, the prevalence, hierarchical depth, and automaticity of use in these domains seem limited compared to language.

Recursion has also been investigated in atypical development. Some studies have investigated the contribution of linguistic recursion to second-order ToM in autism (27, 28). Their results show that recursive syntax (sentential complementation) predicts second-order false beliefs (28) and that training with sentential complementation improves ToM, with most gains for children with mild autism (27). However, this relationship might be limited to explicit ToM and explained by the role of working memory in both domains (28–30).

In line with findings on specific autistic cognition, language development is heterogeneous in autism (31). Highly atypical developmental trajectories have been reported, including cases where missing early communication skills, such as joint attention, do not preclude the emergence of structural language (32). Similarly, literacy skills suggestive of implicit meaning-making have been observed in nonspeaking autistic individuals, offering a potential alternative route to language acquisition (33). As for syntax, syntactic deficits can occur even when vocabulary and sentence repetition are intact (34–36), and 20-30% of individuals with autism are non-verbal or minimally verbal (37). In this subgroup, linguistic recursion is absent, similar to cases in which children were not exposed to language in critical periods (see 38). In the latter, though, fluency and lexical abilities are preserved.

Linguistic comprehension in non-verbal and minimally verbal individuals with autism is vastly understudied. Slušná et al. (39) studied individuals with non- or minimally verbal autism and has found that expressive language (General level of no echolalic language, measured with the ADOS; see **Table 1** for details) correlates with receptive language (or verbal mental age, measured with the Peabody Picture Vocabulary Test–III (PPVT), or with the Clinical Evaluation of Language Fundamentals (CELF) when PPVT score reached a verbal mental age (VMA) of 3 years) but not with non-verbal IQ. This study also shows a strong association between sense-making/ComFor (Forerunners in Communication) and Verbal Mental Age/Receptive Language.

**Table 1:** Summary of (diagnostic) tests applied to A. Testing was done in the context of the Slušná et al. (39) study on non-/minimally verbal individuals with ASD.

Test	Goal	Results	Tasks/Observations
Leiter-3	Non-verbal intelligence	Low rank (CI 79)	Figure placement, model completion, analogy classification, sequential order
Peabody-III	Auditory comprehension of words	Low level (equivalent to a 4.25 year old)	Pointing to visual referents, with simple or abstract meanings
ComFor	Functional and referential representations	Symbolic representation, using visual support	Symbolic comprehension of objects and actions by bidimensional support: photos, drawings, pictograms, with written words
ADOS-2 (module 1)	ASD diagnosis	Classified as ASD: 8 (PD18) Symptomatology level: Moderate (6)	Socio-affective communication, reciprocal social interaction, stereotyped behavior and restricted interests, playfulness
ADI-r (interview)	Complementary clinical ASD diagnosis	Classified as ASD A) Reciprocal social interaction: 27 (>10) B) Communication anomalies: 14 (>8) C) Restricted/repeated stereotyped behavior: 10 (>3) D) Early developmental evidence: 3 (>1)	Lack of direct gaze or social smile, limited use of conventional gestures or spontaneous imitation, circumscribed interests (e.g., object parts) and mannerisms (e.g., with hands and fingers), developmental behavioral anomalies during the first 3 years of life (e.g., facial expressions)

A. reached the representational level in the Communication and Functioning Assessment (ComFor), like most participants, showing that reaching the highest level of visually grounded symbolic representation does not guarantee language access. All the tests were administered by a psychologist specialized in ASD [Andrea Rodríguez Poveda, reg. 21751 Col·legi oficial de psicòlegs de Catalunya], except for the Autism Diagnostic Interview-Revised (ADI-r interview), which was done by a specifically trained doctoral student. The other tests administered were: ADOS-2 (Module 1): Autism Diagnostic Observation Schedule, Second Edition – Module 1; Leiter-3: Leiter International Performance Scale, Third Edition; Peabody-III: Peabody Picture Vocabulary Test, Third Edition (PPVT-III).

In another study, Vicente et al. (40) investigated whether autistic adults with minimally receptive vocabulary could comprehend two-word phrases presented auditorily where a familiar noun is modified by a familiar adjective (e.g., red broom). They found that only one out of eight participants succeeded. Interestingly, the pattern was reversed with a visual version of the same task, as all but one participant could form simple symbolic compositions with iconic representations in all trials. This opens the question of whether visual-spatial recursion could also be available to minimally verbal populations with autism and, more broadly, whether language is necessary for recursion in vision.

Previous work on the relationship between language and visual recursion paints an ambiguous picture. On the one hand, verbal or motor interference does not affect the capacity to perform adequately in a visual recursion task (10). Moreover, when testing well-trained participants with motor, visual, and music recursion, fMRI experiments show domain-specific patterns of brain activity (5, 7, 41). Thus, each domain could implement similar recursive computations on independent biological systems (8, 9). On the other hand, when testing untrained participants, the behavioral and neural evidence is compatible with shared resources in acquiring recursive rules across domains (6, 42, 43). For instance, stroke patients with deficits in parsing sentences with two levels of embedding are also impaired in the processing of visual recursion, and visual recursion seems to activate a left-lateralized brain network, including the classic language brain areas (42, 43). However, these studies were performed in adults with fully developed language.

Perhaps recursion is domain-general at first, but acquires specializations depending on the degree of exposure and training with specific domains. The adult neural pattern associated with processing natural and artificial languages only emerges after extensive training and in specific development stages (44–47). More extensive exposure and training could explain why recursion is expressed first and more prevalently in language than in other domains. However, recursion might also become available later to other domains.

In our previous work in the visual domain, we found that children could master visual recursion around age 9 but not before (48). This is later than the acquisition of syntax. However, the age of acquisition of recursion may vary across linguistic forms, being available for simple adjectives and verbal compounds at age 2–3 and more complex sentence complements at age 5-7 (49). We also found that prior acquisition of a simpler iterative task was necessary to master recursion within the same developmental stage (48). This effect resembles the acquisition constraints in language, in which conjunctive constructions must be mastered before recursion (50, 51). In conjunction and iteration, items are added within a fixed hierarchical level without generating new levels (e.g., very, very, very big).

Interestingly, we also found that grammar comprehension (controlling for IQ) equally predicted accuracy in visual recursion and iteration. However, these grammar comprehension tasks included sentences with a maximum of one embedded clause. Unlike our study with stroke patients mentioned above, they were not designed to test for the similarities between linguistic and visual recursion (42).

This manuscript explores the relationship between visual and syntactic recursion from a developmental perspective by presenting a case report from an 11-year-old child (A.) with autism and hyperlexia (mechanical reading), whose linguistic skills do not exceed those of minimally verbal individuals despite possessing a visually-based, predominantly nominal vocabulary acquired by reading. We investigated whether visual recursion can dissociate from linguistic syntax by testing A. with a visual recursion task and a control visual iteration task. We compared his accuracy levels with 26 Typical Development (TD) children attending the 4th grade. If linguistic recursion is a prerequisite for visual recursion, we would expect A. to fail in the former. However, he could still succeed in the control iteration task due to his preserved visual cognitive performance. Conversely, if recursion in vision is not dependent on language, we expect A. to pass both recursion and iteration tasks.

## Methods

### Case report

A. is a minimally verbal 11-year-old child with autism and hyperlexia. Crucially, a minimally verbal condition includes a lack of language comprehension. A.’s language knowledge consists of absent spontaneous production and a receptive vocabulary biased towards nouns (and a few adjectives) corresponding to objects (and properties) that can be represented in pictures. His language abilities appeared for the first time at the age of 2.6 y.o. in the form of precocious oral reading of words as captions of object pictures presented in cards. His speech is infrequent and, when not echolalic, comprises a maximum of two-word combinations for requests (e.g., ‘batido rosa’, which means ‘pink shake’ in English). A slower and over-articulated simplified Spanish has spontaneously emerged in his family to ensure that A. understands the behavioral regulatory instructions that the linguistic exchange with him mainly consists of. In this family code, verb and noun grammar is kept to a minimum, wh-questions are replaced with simplified alternative questions (e.g., *A., pear, or banana*)?, and no pronouns exist. Like his minimally verbal (and nonverbal) peers, A. has neither declarative pointing nor iconic gestures. Unlike them, he does not use alternative augmentative communication systems (e.g., PECS, Picture Exchange Communication System).

A.’s score on the Peabody Picture Vocabulary Test (PPVT-III), which yields a verbal mental age of 4.3 years, stems from his proficiency in labeling pictures with nouns, thus clearly overstating his linguistic knowledge. In contrast, 4-year-olds produce and understand complex utterances and are active conversationalists. Moreover, previous research has shown that while PPVT vocabulary knowledge scores and verbal mental age correlate well in normal development, this correlation is less clear in autism (52).

Hyperlexia occurs when reading decoding skills surpass reading comprehension (53, 54). While autism and hyperlexia are strongly associated (53), the combination of hyperlexia and minimally verbal autism is exceptional; it has only been described once in the literature, but in a much younger autistic child of 4 years of age (see 55). Hyperlexia-based interventions in verbal autism seem to improve comprehension but not expressive language (56), which aligns with A.’s extremely poor expressive language despite hyperlexia allowing him to fluently read aloud in Spanish, Catalan, and English.

A. received his autism spectrum disorder diagnosis at Sant Joan de Déu Hospital in Barcelona (a children’s hospital) at the age of two. An overview of his assessment is shown in **Table 1**. His gross motor milestones were achieved at the lower end of the normal age range, while his fine motor skills, influenced by hand hypotonia and palm hypersensitivity, proved atypical. Although A. can respond to joint attention, initiating it is challenging. He was evaluated with the ADI-r (Autism Diagnostic Interview-revised), the ComFor (a test probing which augmentative communication system is more appropriate for the tested individual, 57), and the Leiter-3 (Leiter International Performance Scale, measuring non-verbal intelligence). Notably, A. demonstrated deficits in social interaction (27; cutoff 10), communication (14; cutoff 8), restricted and repetitive behaviors (10; cutoff 3), and atypical development detectable by 36 months or before (3; cutoff 1), based on ADI-r scoring, consistent with his ASD diagnosis. A. reached the representation level in the scale of “perception and sense-making of non transient forms of communication” measured by the ComFor (58). This score is similar to his nonverbal/minimally verbal peers. On Leiter-3’s subtests assessing fluid intelligence, A. scored 79 IQ points. In the specific subtasks, he scored 5 in Figure-Ground, a visual interference task where the target object is embedded in an increasingly complex background; 5 in Form Completion, where the subject must arrange parts in a whole; 8 in Classification and Analogies, which tests pattern analysis and prediction of “what goes next”; and 6 in the capacity to analyze Sequential Order (59).

Finally, A. has a remarkable ability to type and copy colorful computer displays. Thanks to his familiarization with computers and screens, we tested him with a tactile screen presenting the visual recursion and iteration tasks.

### Visual recursion task (REC) and visual iteration task (ITE)

**Figure 1:**
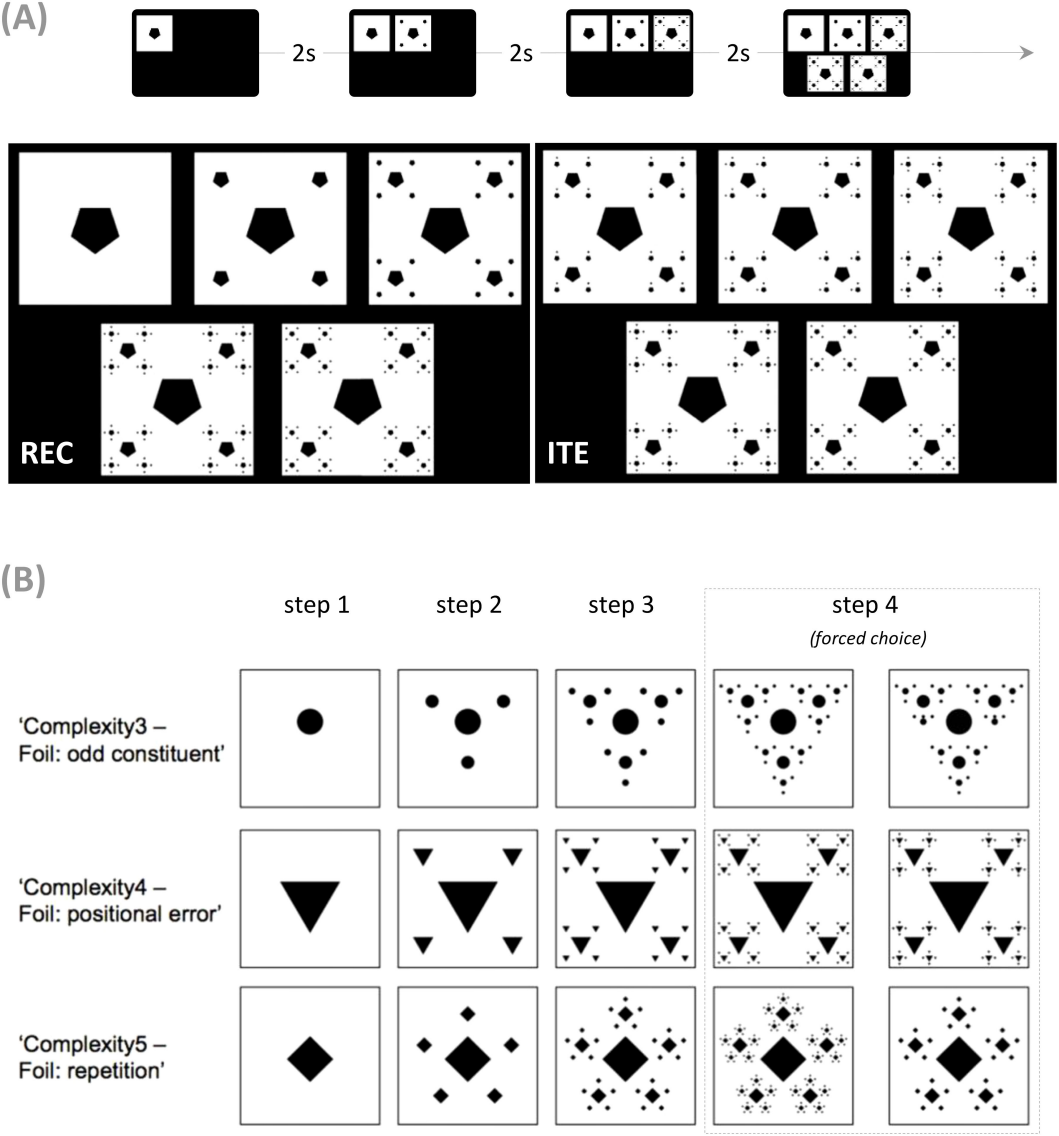
Experimental paradigm [adapted from (42)]. **(A)** The presentation of the visual recursion/iteration task (REC, ITE) comprised 4 steps, including a successive presentation of steps 1 to 3 at the top of the screen, followed by the two options for a forced choice at the bottom. The location of the correct image was randomized and counterbalanced (e.g., left in the ITE and right in the REC example provided). **(B)** Examples of fractals used in REC. There were different categories of ‘visual complexity’ — 3, 4, and 5 — and different categories of foils (the task comprised three trials of each of the nine categories). In ‘odd constituent’ foils, two elements within the whole hierarchy were misplaced —foil, second panel step 4; in ‘positional error’ foils, all elements within new hierarchical levels were internally consistent but inconsistent with the previous iterations —foil, second panel step 4; in ‘repetition’ foils, no additional iterative step was performed after the third iteration —foil, second panel step 4.

In the visual Recursion task (REC) (**Figure 1A**), participants are shown a sequence of three images (steps 1, 2, and 3) depicting the generation of a visual fractal and asked to discriminate, from two choices, the image corresponding to the correct continuation of the previous sequence of three (i.e., the fourth step). One of the choices is the correct image, and the other is a foil (**Figure 1B**). Stimuli varied along two categories, each with three levels: visual complexity (the number of increasing elements: 3, 4, or 5) and type of foils. The latter could be ‘odd constituent’ foils (two elements within the whole hierarchy were misplaced), ‘positional error’ foils (all elements within new hierarchical levels were internally consistent but inconsistent with the previous iterations), and ‘repetition’ foils (no additional iterative step was performed after the third iteration). Combined, these two categories generated 9 different kinds of stimuli. The tasks comprised three instances of each of these nine kinds, resulting in 27 trials.

Participants presented their answers by touching their choice directly on the screen within a maximum of 30 seconds, after which there was a trial timeout. No response feedback was given. The total duration of the task (27 trials) was about 12 minutes. The task is an adaptation of what is used and described in detail elsewhere (5, 42, 48). The task is well-validated and designed to prevent the use of simple visual strategies (22). For example, choosing the image more similar to step 3 would yield incorrect answers as the repetition foil is identical to the latter. In previous work, we found that low-frequency visual information was insufficient to determine the correct choice (10), rendering simple Gestalt-based strategies unlikely. Finally, we found that REC’s scores correlate with correctly representing hierarchical information in the musical, action, linguistic, and logic domains when the variance shared with simple iteration is accounted for (6, 42, 43).

The control task, the visual Iteration task (ITE), also involved a stepwise procedure applied to hierarchical structures without recursive embedding. However, in ITE, additional elements are added to a preexisting hierarchical structure without producing new hierarchical levels (**Figure 1A**, bottom right). As for REC, understanding this stepwise procedure is necessary to predict the next step correctly.

### Procedure

The procedure comprised one training session and two test sessions. In the training session, comprised of 20 trials, A. was habituated to the touchscreen apparatus and tested with simple (non-recursive) geometrical reasoning tasks. Similarly to REC and ITE, he learned to follow the consecutive presentation of three figures on the upper part of the screen and touch the correct figure from the two alternatives at the bottom. The trials provided success or error auditory feedback. In the first 8 trials, he was asked to identify the identity of geometric shapes (e.g., three circles on the top row, then a circle *vs*. a square in the bottom row). In the following 12 trials, he had to grasp how a pattern was incrementally built from an initial image in the top row consisting of one central big circle alone (4 trials), one big and one small circle (4 trials), or one big and two small circles (4 trials). Each initial arrangement was sequentially incremented in the top row, yielding 1, 2, 3 circles; 2, 3, 4 circles; and 3, 4, 5 circles, respectively. The participant then had to choose between the two bottom images: the correct one with the congruent increment (i.e., 4 circles, 5 circles, and 6 circles, respectively) and the incorrect one with no increment (i.e., repeating the third occurrence of the top row).

Feedback sounds were removed from the test sessions. Five months later, the first test session was performed, which comprised ITE and REC. Three months later, the second session comprised REC, followed by ITE, presenting the stimuli in a different order.

Videos of the testing sessions will be available on demand with the permission of A.’s parents.

We compared A.’s performance to 26 children with Typical Development (TD) (11 female, ages 9-10, 4th-grade monolingual students from Austria). These children were tested with REC and ITE in a counterbalanced order (13 children ITE-REC and 13 REC-ITE) and were part of a previous study (48).

We analyzed and visualized the recorded data using Jamovi (60), Matlab (61), and Raincloud plots (62).

## Results

### Accuracy

**Figure 2:**
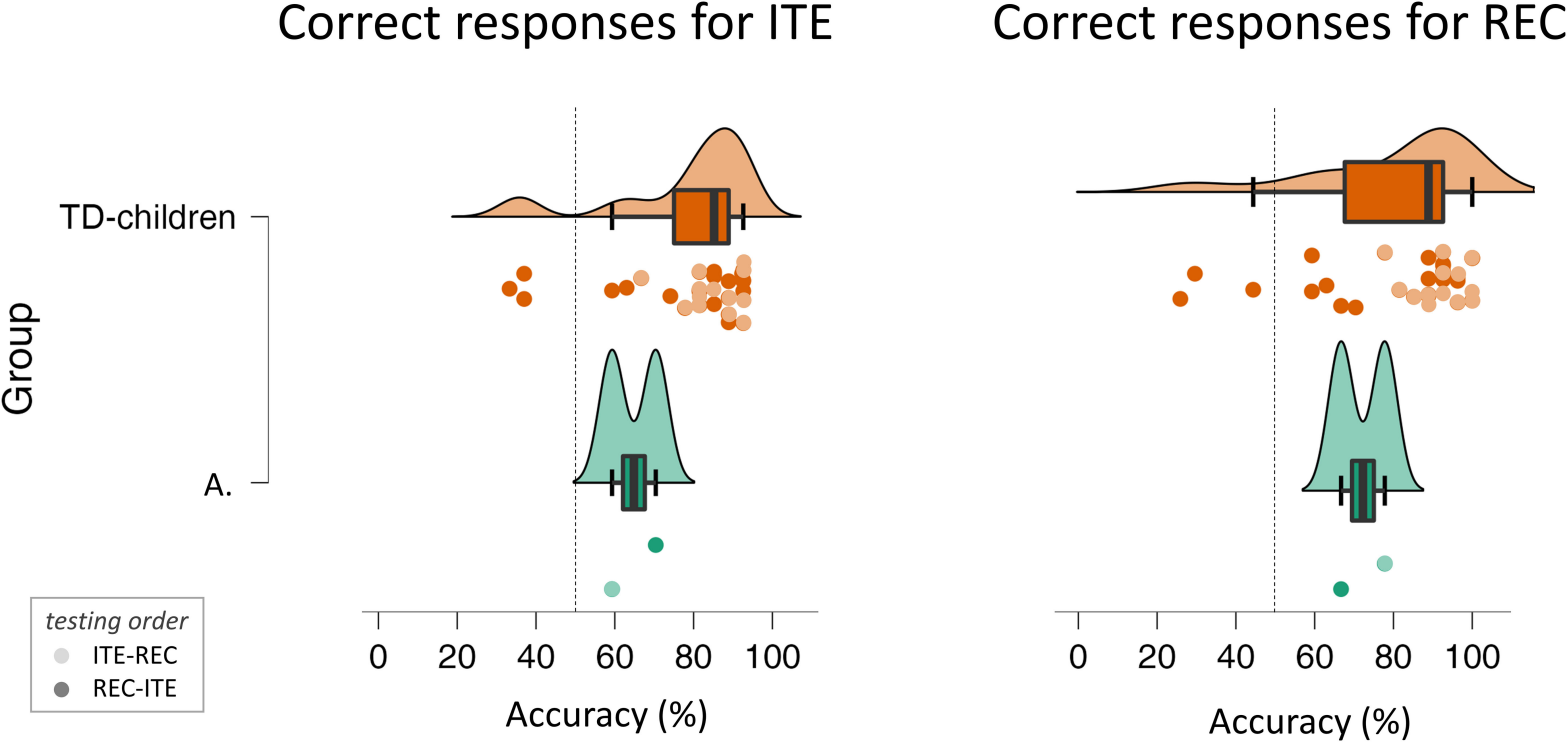
Percentage of correct responses given by A. and the children with Typical Development (TD). Both groups were tested with the visual recursion task (REC) and the visual Iteration task (ITE), with counterbalanced order. A.’s first session order was ITE-REC (light color), and the second session was REC-ITE (dark color). The dashed line marks 50% accuracy as the chance level..

In the first test session, A. hesitated to answer the initial 10 ITE trials and timed out after 30 seconds (see Reaction Times in **Supplementary Figure S1**). We labeled these trials as “incorrect” in terms of accuracy to apply a stringent criterion. Across both sessions, ITE accuracy was 64.8% and REC 72.2%, both above chance (ITE binomial GLM intercept: z = 9.97, *p* <.001; REC: z = 11.80, *p* <.001) (**Figure 2**). There were no significant associations between correct answers and the order of each session, neither for ITE (first session 59.3%; second session 70.4%, chi-square test of independence: χ ^2^ (1,54) = 0.73, *p* = .393) nor for REC (first session 77.8%, second session 66.7%; χ ^2^ (1, 54) = 0.83, *p* = .362). These results indicate that A. performed adequately and equally well in REC and ITE.

### Comparison with children with typical development

A.’s mean accuracy did not differ from children with TD of similar age, neither in ITE (64.8 ± 7.8%, *vs*. 77.9 ± 18.0%; independent sample t-test: t_(26)_ = -1.007, *p* = .323), nor in REC (72.3 ± 7.8% *vs*. 79.6%, ± 21.2%; t_(26)_ = -0.482, *p* = .634). Following the procedure in Huber et al. (63) to compare case reports with control groups, we ran a Generalized Mixed Model to ascertain the effects of Task (REC, ITE), Group (A., children with TD), and their interaction on the likelihood of correct responses (1/0). As random effects, we included Task: ID and 1:Trial. Confirming the t-test analysis, we found neither differences between groups (B = 0.8, 95% CI [0.28, 18.2], z= 0.75, *p*= .45), tasks (B = 0.33, 95% CI [0.71, 2.76], z= 0.96, *p*= .34), nor an interaction between group and task (B = -0.04, 95% CI [0.24, 3.75], z= -0.06, *p*= .95).

While we could not compare the reaction times between A. and the children with TD due to A. multitasking behavior (see the comments and data visualizations in **Supplementary Materials Figure S1**), we were able to explore the effects of presentation order of the tests. As in Martins et al. (48), the tests showed that the order ITE-REC (i.e., iteration test first, recursion test second) may have benefited children with TD (see **Supplementary Materials Table S1**), but not A., who performed slightly worse in the first session with the ITE-REC order (see **Supplementary Materials; Figures S2, S3**; **Tables S2, S3**).

## Discussion

We presented a case report of an 11 y.o. minimally verbal but hyperlexic autistic child who can represent visual recursion despite showing no evidence of this capacity in linguistic production or comprehension. His accuracy was above chance and was not significantly different from that of 9–10 y.o. children with TD. This suggests that visual recursion is not dependent on a primary linguistic recursive capacity but could be domain-general or multiply domain-specific.

These findings resonate with prior research on autistic cognition, which shows marked dissociations between verbal and nonverbal intelligence (64). Many minimally verbal autistic individuals demonstrate significantly higher scores on nonverbal reasoning tasks such as Raven’s Progressive Matrices despite low scores on verbal subtests of traditional IQ measures. A.’s case similarly suggests that the capacity for recursion may be preserved in visuospatial domains while remaining inaccessible in language.

Within the generative framework (65), language recursion is the core combinatorial capacity that builds an “infinite array of expressions” that are interpreted at two interfaces, a conceptual-intentional (C-I) system connecting the same mental expressions to reasoning and planning among “other activities of the internalized mental world” and a sensorimotor (S-M) system connecting with the external world via language production and perception (externalization). We surmise that A.’s impairment in linguistic recursion might result from deficits in both the C-I and the S-M systems required for language, which may prevent recursion to manifest linguistically.

Visual recursion is likely to hinge on different machinery. First, it does not involve an intentional component. Second, it has a sensory component but does not involve a structured motor output like linguistic utterances. Thus, while language recursion requires intact loops involving core, conceptual-intentional, and sensorimotor systems, visual recursion can operate with core and visual sensory and conceptual systems without intentional and motor components. As reviewed above, visual conceptual and combinatorial properties might be preserved in minimally verbal autism (40).

This interpretation remains agnostic regarding whether recursion is domain-general or multiply domain-specific (10). However, it hypothetically (but not necessarily) allows the same core capacity to interface with specialized sensorimotor/sensory and conceptual-intentional/conceptual systems. In this regard, A.’s hyperlexia suggests a strong visual sensory system and the ability to associate visual patterns with vocal labels but without linguistic semantics. Interestingly, although his visual IQ was low in most subtasks in the Leiter-3, he had a medium-range score in the subtest Classification and Analogies, which explicitly assesses the capacity for general pattern analysis and prediction of “what goes next”. The latter can indicate a capacity for recognizing abstract visual patterns and rules (i.e., a visual conceptual system).

In sum, specialization of the sensory and conceptual abilities to the visual domain could afford the interaction of these systems with a core combinatorial capacity and the detection of visual recursion. Conversely, a faulty integration of the subcomponents making the C-I and S-M systems would impede expressive and receptive recursion in language.

This study has two significant limitations. First, broader generalizations are impossible, as this case report presents a single subject with a rare profile: he is a minimally verbal autistic child with hyperlexia in 3 languages. Second, we did not directly test A. with a linguistic recursion comprehension task. As such, we do not know whether A. could demonstrate recursion in a reading or writing task, should he be taught to read or write. Recent evidence suggests that nonspeaking autistic adolescents and adults may display implicit knowledge of English orthography in response time-based spelling tasks, suggesting foundational literacy skills (33). However, it is unclear whether this implicit capacity for orthography translates into semantic and, more importantly, syntactic understanding (66). Here, we infer limited syntactic recursion from A.’s limited expressive compositionality (i.e., two-word phrase requests at most; e.g., *pink milkshake*) and his low (and likely overestimated) score on the vocabulary test PPVT. Since previous research has shown an association between receptive and expressive language (39, 52) and very limited phrase comprehension in minimally verbal autism (40), we adduce that A. was also limited in linguistic recursion.

Finally, it is important to acknowledge that a lack of functional language might stem from impairments at either abstract computational abilities, such as recursion, or from an absence of verbs and/or grammar, which are fundamental and specific to language. Our results show that recursion is present, at least in vision, albeit possibly made available by simpler domain-specific machinery. However, it is also possible that a domain-general system is not available to language due to the latter set of limitations.

## Data Availability

The datasets presented in this study can be found in online repositories. The names of the repository/repositories and accession number(s) can be found below: https://osf.io/aju83/.
