# Supplementary material for: Visual recursion without recursive language? a case study of a minimally verbal autistic child
Source: Front Psychiatry. 2025 Jun 23;16:1540985. doi: 10.3389/fpsyt.2025.1540985 (PMC12232466; doi:10.3389/fpsyt.2025.1540985)
Supplement: Supplementary file 1 [file DataSheet1.pdf]

Visual recursion without recursive language? a case study of a minimally verbal autistic child

SUPPLEMENTARY MATERIALS

JOANA ROSSELLÓ[*1+], ALEXANDRE CELMA-MIRALLES[2,3+], MAURICIO DIAS MARTINS[4]

[*]Corresponding Author: joana.rossello@ub.edu

+These authors contributed equally to this work.

[1]Dpt. of Catalan Philology and General Linguistics, University of Barcelona, Spain
[2] Center for Music in the Brain, Aarhus University & Royal Academy of Music Aarhus/Aalborg, Aarhus, Denmark
[3]Center for Brain and Cognition, Pompeu Fabra University, Barcelona, Spain
[4]SCAN-Unit, Department of Cognition, Emotion, and Methods in Psychology, Faculty of Psychology, University of Vienna, Austria

Front. Psychiatry - Sec. Autism - Volume 16 - 2025 | doi: 10.3389/fpsyt.2025.1540985

1. Reaction Times of A. responses and TD-children

The reaction times of all the responses (including time-out responses) by A. and TD-children are depicted for each test in Figure S1. Because A. responses often timed out after 30 s, we describe RTs averages including those "timeouts" to appropriately compare A. and TDs responses. The mean RTs for A. were: ITE-1st: 6.6s, ITE-2nd: 3.8s, REC-1st: 2.8s, REC-2nd: 6.9s. For TDs RTs were: ITE-1st: 7.8s, ITE-2nd: 8.3s, REC-1st: 8.8s, REC-2nd: 6.5s. Looking at the RT distribution in Figure S1, A. seems to give responses during the first 15 seconds, or otherwise, he does not give any response at all. This contrasts with the TD-children who give responses later on, which may reflect some reasoning processes and hesitations (see Figure S1). It is important to note that A's attention was difficult to control because videos were playing on the phone on the second day of testing, and he sometimes got up and ran away on the first day of testing. Thus, any statistical comparison of RTs would not directly inform about the speed of the computation of iteration or recursion in the brain due to these two confounds completely absent in TD-children.

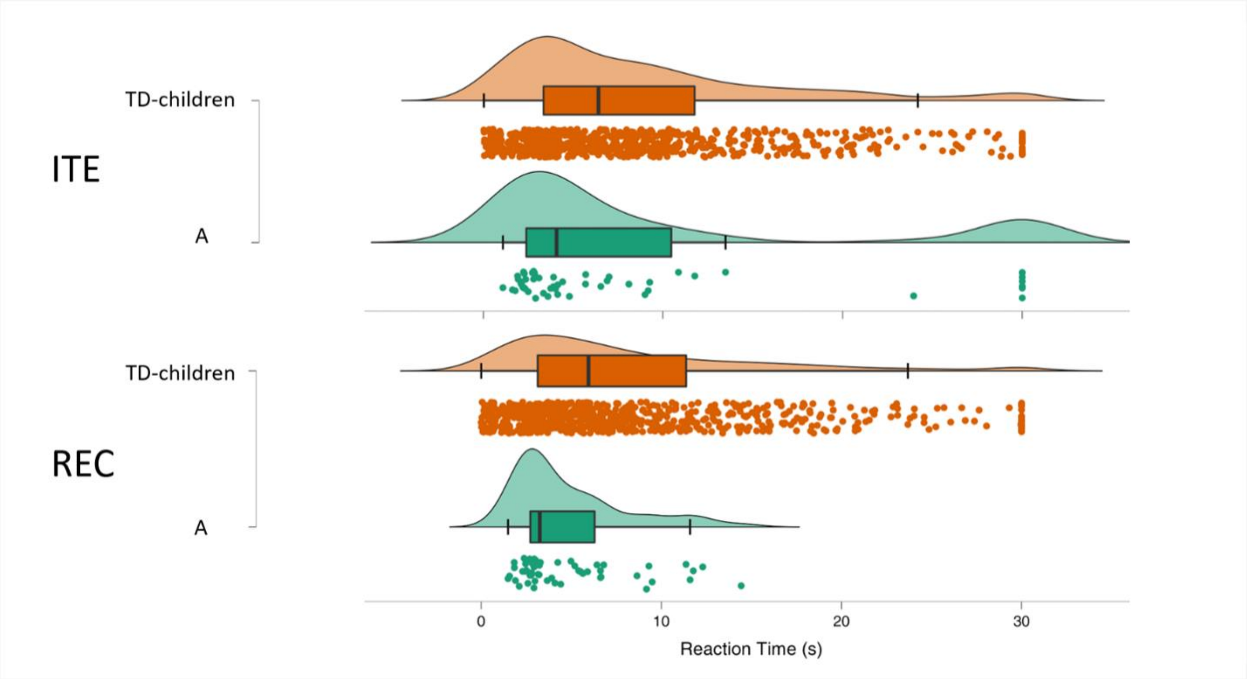

Figure S1. Reaction Times of A. and TD-children responses for each trial. Notice that time-out responses appear here, represented at 30 seconds.* *We ran a linear model to predict RT (excluding outliers) using three predictors (and their interactions): Correct responses (1, 0), Task (ITE, REC), and Position in the procedure (First, Second). The model was a better fit than a null model $F(7) = 4.76$, $p < .001$, and explained 21 % (adjusted R^2) of the variance. However, no predictors nor interactions were significant (all $p > .05$).*

2. Testing order effect on TD-children

A Mixed Design Anova (within-factor "test": ITE, REC; between-factor "order": ITE-REC, REC-ITE) shows that the TD-children had higher accuracy in the REC test when it was performed after the ITE test (i.e., testing order ITE-REC). Specifically, the accuracy for REC in the second position was higher than that for REC in the first position and ITE in the second. This effect of the task order is in line with the results reported by the General Estimating Equations model by Martins and colleagues (2014).

Within Subjects Effects

Cases	Sum of Squares	df	Mean Square	F	p
Test	37.911	1	37.911	0.587	0.451
Test ✻ Order	341.197	1	341.197	5.279	0.031
Residuals	1551.182	24	64.633		

Note. Type III Sum of Squares

Post Hoc Comparisons - Order

		Mean Difference	SE	t	p_{bonf}		p_{holm}	
(ITE-REC)	(REC-ITE)	19.092	6.391	2.987	.006	**	.006	**

** p < .01 *Note.* Results are averaged over the levels of: Test

Post Hoc Comparisons - Order ✻ Test

		Mean Difference	SE	t	p_{bonf}		p_{holm}	
(ITE-REC, ITE)	(REC-ITE, ITE)	13.969	6.769	2.064	.287		.121	
	(ITE-REC, REC)	-6.831	3.153	-2.166	.243		.121	
	(REC-ITE, REC)	17.385	6.769	2.568	.093		.062	
(REC-ITE, ITE)	(ITE-REC, REC)	-20.800	6.769	-3.073	.027	*	.023	*
	(REC-ITE, REC)	3.415	3.153	1.083	1.000		.290	
(ITE-REC, REC)	(REC-ITE, REC)	24.215	6.769	3.577	.007	**	.007	**

* p < .05, ** p < .01 *Note.* P-value adjusted for comparing a family of 6

Tables S1. Results of the Mixed-Design ANOVA with TD-children and post hoc comparisons.

3. Testing order effect on TD-children versus A. (one-sample t-tests)

To explore any difference depending on the order of the tests, we ran four two-tailed one-sample *t*-tests, in which TD-children's accuracy was compared against A.'s accuracy. Parametric and non-parametric tests were applied depending on the Shapiro-Wilk test for normality.

For the testing order, ITE-REC, A.'s accuracy in ITE (M = 59.3%) was significantly lower than TD-children's accuracy (M = 84.9%, SD = 7.6, Mdn =85.2%, Wilcoxon = -91, p = .002). Similarly, A.'s accuracy in REC (M = 77.8%) was significantly lower than TD-children's accuracy (M = 91.7%, SD = 7.1, $t_{(12)} = -7.073$, $p < .001$). These findings remain significant if the alpha level is Bonferroni-corrected for multiple comparisons (i.e. $p/4 = .0125$). In contrast, for the testing order REC-ITE, A.'s accuracy in the ITE test (M = 70.4%) was not significantly lower than TD-children's accuracy (M = 70.9%, SD = 22.6, Mdn = 85.2%, Wilcoxon = -50.0, p = .779). Neither A.'s accuracy in the REC test (M = 66.7%) was significantly lower than TD-children's accuracy (M = 67.5%, SD = 23.9, $t_{(12)} = -0.125$, p = .902).

These results suggest that A. performed worse than the TD-children in his first session, when the tests followed the order ITE-REC, or that TD-children generally performed better when the ITE test preceded the REC test. Indeed, children benefited from this testing order with higher correct scores in the REC test (see previous Mixed Design Anova), while A. did not (see next Generalized Mixed Model). Together, this shows that A.'s REC accuracy does not vary with task order, in contrast to TD-children, who benefited from previous experience with the ITE. Thus, A. differed from TD-children in performing more uniformly across the tests.

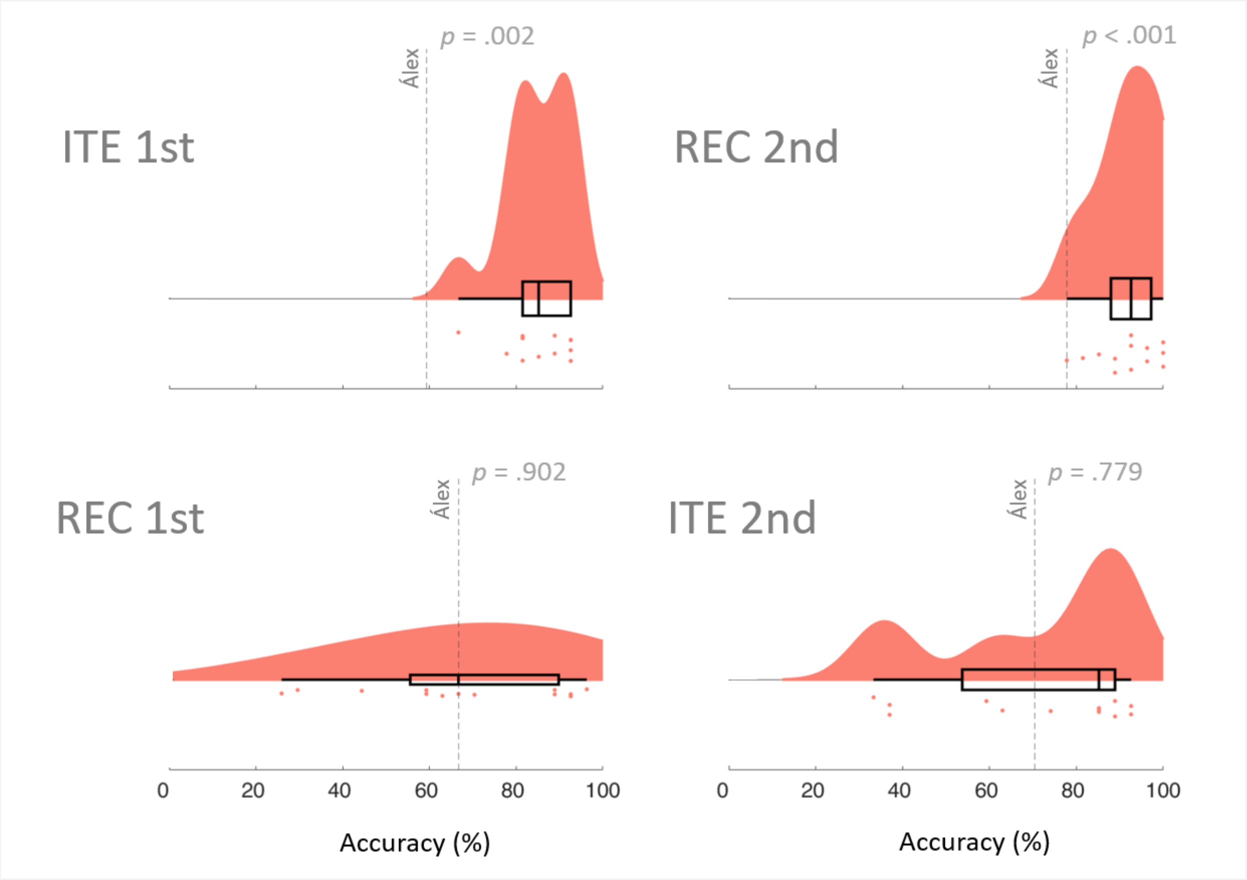

Figure S2. Two-tailed one-sample *t*-tests comparing the response accuracy of TD-children (rainclouds) against the response accuracy of A. (dashed line). A.' responses were generally lower in the testing order ITE-REC (first row), corresponding to his first session, while TD-children performed better in REC 2nd.

4. Testing order effect on both TD-children and A. (Generalized Mixed Model)

To compare A. and TD-children, we run a Generalized Mixed Model to ascertain the effects of Task (REC, ITE), Position (First, Second), and Group (A., TD-children) on the likelihood of correct responses (1/0). As fixed effects, we included all the main effects and interactions. As random effects, we included Task:Name and TrialOrder:Task. The model explained 26.4 % (conditional R^2) of the variance. We found significant effects of Position, Task*Position, and Task*Group*Position (see Supplementary Table 2).

Post hoc comparisons were performed for the triple interaction with Holm *p*-value correction. They revealed significant differences between the REC presented in the second position vs. 1) the ITE presented in the second position and 2) the REC presented in the first position (see Supplementary Table 3), but only for the TD-children. This shows that A.'s REC accuracy does not vary with task order, in contrast to TD-children (Figure S2), who benefited from previous experience with the ITE.

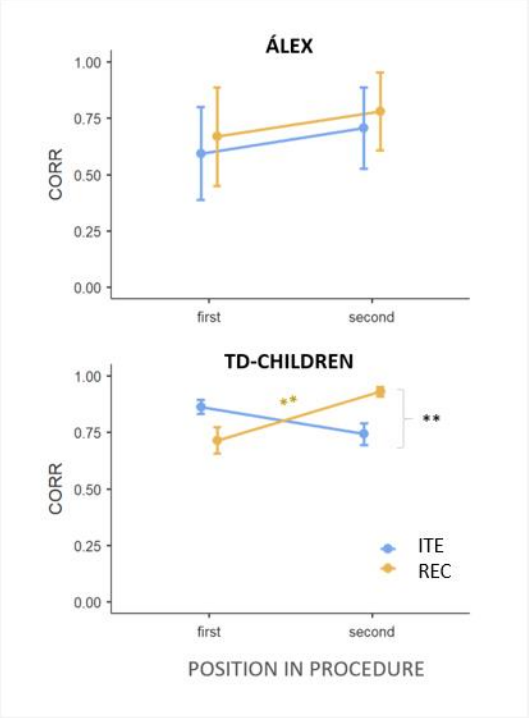

Figure S3. Estimated means and standard errors of correct responses for A. and the TD-children depending on the task (REC, ITE) and its position in the procedure order (First, Second). TD-children had significant increases for the REC in the second position compared to (i) ITE in the second position and (ii) REC in the first position.

Fixed Effects Parameter Estimates

				95% CI				
Names	Effect	Estimate	SE	Lower	Upper	exp(B)	z	p
(Intercept)	(Intercept)	1.2129	0.438	0.3554	2.070	3.363	2.772	0.006
task1	REC - ITE	0.3074	0.271	-0.2235	0.838	1.360	1.135	0.256
group1	TD-Children - A.	0.7972	0.872	-0.9117	2.506	2.219	0.914	0.361
position_in_procedure1	second - first	0.4886	0.228	0.0424	0.935	1.630	2.146	**0.032**
task1 ✻ group1	REC - ITE✻ TD-children - A.	-0.0717	0.526	-1.1035	0.960	0.931	-0.136	0.892
task1 ✻ position_in_procedure1	REC - ITE✻ second - first	1.2582	0.559	0.1636	2.353	3.519	2.253	**0.024**
group1 ✻ position_in_procedure1	TD-children - A. ✻ second - first	-0.0969	0.455	-0.9892	0.795	0.908	-0.213	0.831
task1 ✻ group1 ✻ position_in_procedure1	REC - ITE✻ TD-children - A. ✻ second - first	2.3999	1.117	0.2108	4.589	11.022	2.149	**0.032**

Table S2. Results of the Generalized Mixed Model for correct responses, depending on Group, Task and Position in procedure, and all their interactions. Significant *p*-values and estimates are marked in bold and red color (respectively), and highlighted in yellow.

Post Hoc Comparisons - task ✻ grade ✻ position_in_procedure

task	group	position_in_procedure		task	group	position_in_procedure	Difference	SE	z	p_{holm}
ITE	A.	second	-	ITE	TD-children	second	0.831	0.7645	-0.2008	1.000
ITE	A.	second	-	REC	A.	second	0.689	0.4715	-0.5443	1.000
ITE	A.	second	-	REC	TD-children	second	0.185	0.1748	-1.7873	1.000
ITE	A.	first	-	ITE	A.	second	0.602	0.3515	-0.8695	1.000
ITE	A.	first	-	ITE	TD-children	second	0.500	0.4536	-0.7638	1.000
ITE	A.	first	-	ITE	TD-children	first	0.227	0.2068	-1.6280	1.000
ITE	A.	first	-	REC	A.	second	0.415	0.2764	-1.3205	1.000

Post Hoc Comparisons - task ✻ grade ✻ position_in_procedure

task	group	position_in_procedure		task	group	position_in_procedure	Difference	SE	z	p_{holm}
ITE	A.	first	-	REC	A.	first	0.730	0.4604	-0.4984	1.000
ITE	A.	first	-	REC	TD-children	second	0.112	0.1038	-2.3575	0.442
ITE	A.	first	-	REC	TD-children	first	0.592	0.5431	-0.5713	1.000
ITE	TD-children	second	-	REC	TD-children	second	0.223	0.0930	-3.5964	**0.009**
ITE	TD-children	first	-	ITE	A.	second	2.647	2.4427	1.0552	1.000
ITE	TD-children	first	-	ITE	TD-children	second	2.201	0.8041	2.1598	0.708
ITE	TD-children	first	-	REC	A.	second	1.824	1.9031	0.5763	1.000
ITE	TD-children	first	-	REC	TD-children	second	0.491	0.1336	-2.6143	0.232
ITE	TD-children	first	-	REC	TD-children	first	2.605	1.0257	2.4321	0.375
REC	A.	second	-	ITE	TD-children	second	1.207	1.2552	0.1805	1.000
REC	A.	second	-	REC	TD-children	second	0.269	0.2844	-1.2420	1.000
REC	A.	first	-	ITE	A.	second	0.824	0.5347	-0.2985	1.000
REC	A.	first	-	ITE	TD-children	second	0.685	0.6969	-0.3719	1.000
REC	A.	first	-	ITE	TD-children	first	0.311	0.3175	-1.1440	1.000
REC	A.	first	-	REC	A.	second	0.568	0.3514	-0.9146	1.000
REC	A.	first	-	REC	TD-children	second	0.153	0.1580	-1.8161	1.000
REC	A.	first	-	REC	TD-children	first	0.811	0.8293	-0.2051	1.000
REC	TD-children	first	-	ITE	A.	second	1.016	0.9451	0.0173	1.000
REC	TD-children	first	-	ITE	TD-children	second	0.845	0.1804	-0.7895	1.000
REC	TD-children	first	-	REC	A.	second	0.700	0.7322	-0.3408	1.000
REC	TD-children	first	-	REC	TD-children	second	0.188	0.0801	-3.9281	**0.002**

Table S3. Post-hoc comparisons for the significant triple interaction Task*Group*Position in procedure of the General Linear Model. Significance level was corrected for multiple comparisons using Holm method for alpha-adjustment. Significant *p*-values are marked in bold and highlighted in yellow.
